# Diagnosis of Brachial Artery Thromboembolism with Point-of-care Ultrasound

**DOI:** 10.5811/cpcem.2019.1.41181

**Published:** 2019-01-22

**Authors:** Vincent Ceretto, Michael Lu

**Affiliations:** University of Rochester Medical Center, Department of Emergency Medicine, Rochester, New York

## CASE PRESENTATION

A 77-year-old male with a history of ventricular bigeminy presented to the emergency department complaining of arm tingling, pain, and poikilothermia. This occurred immediately after the patient reached to use the television remote control device. His right forearm and hand were dusky, cold, pulseless, and had delayed capillary refill compared to the left arm. Strength was intact but light touch sensation was decreased. The emergency physician (EP) performed a point-of-care ultrasound, which showed an occlusive distal brachial, proximal ulnar, and proximal radial artery thrombus (Image, Video). Vascular surgery was consulted and within two hours of arrival the patient was in the operating room without any additional vessel imaging. An embolectomy was performed using a Fogarty catheter. Arterial flow was restored, the hand was revascularized, and the patient’s symptoms resolved. A transesophageal echocardiogram was performed postoperatively, which showed a left atrial appendage thrombus. He was started on lifelong anticoagulation and discharged from the hospital.

## DISCUSSION

Acute upper limb ischemia (AULI) is a rare condition that results from sudden loss of blood flow to an extremity. An EP must rapidly diagnose this condition to prevent limb loss and death. Emboli most commonly originate in the heart as a result of atrial fibrillation, and predominantly embed in the brachial artery (68%).^1^,^2^ Overall, the incidence of thromboembolectomy as a result of AULI was 3.3 per 100,000 person-years among men and 5.2 per 100,000 person-years among women.^1^ Although the diagnosis is made clinically by a detailed history and physical exam (six Ps: paresthesia, pain, pallor, pulselessness, poikilothermia, and paralysis), preoperative imaging is usually sought out. In a study of 182 patients with peripheral artery emboli, surgical and survival outcomes were equivalent when comparing duplex ultrasonography alone to contrast angiography or computed tomography angiography.^3^ Ultrasound offers the advantage of being noninvasive, inexpensive, radiation-free, readily available to EPs, and provides dynamic information about perfusion.

**CPC-EM Capsule**

What do we already know about this clinical entity?

*Acute upper limb ischemia is a rare condition that results from sudden loss of blood flow. Emergency physicians (EPs) must rapidly diagnose this condition to prevent limb loss and death*.

What is the major impact of the image(s)?

*Point-of-care ultrasonography can easily be used by EPs to expedite diagnosis and management*.

How might this improve emergency medicine practice?

*The images provided demonstrate how to identify a brachial artery thrombus*.

## Supplementary Information

Video Point-of-care duplex ultrasound demonstrating an upper extremity brachial artery thrombus.

## Figures and Tables

**Image:**
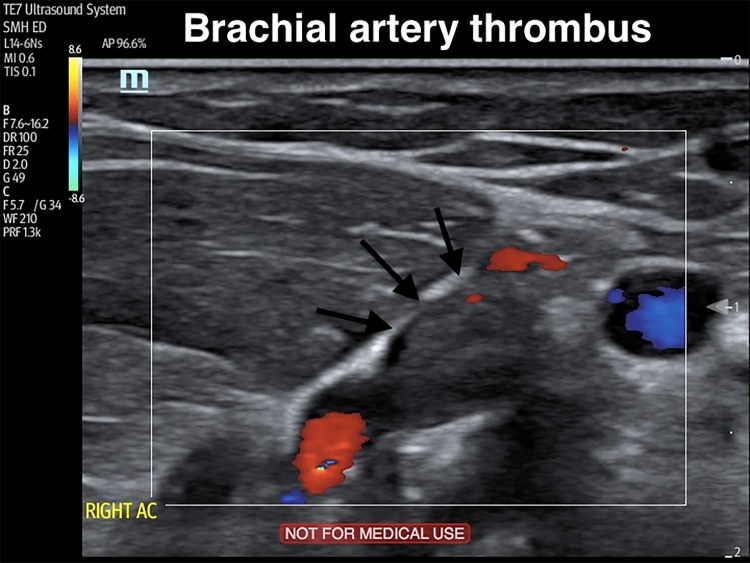
Still image of a duplex ultrasound demonstrating a brachial artery thrombus (arrows).
